# Naltrexone-induced acute generalized exanthematous pustulosis

**DOI:** 10.1016/j.jdcr.2023.07.009

**Published:** 2023-08-03

**Authors:** Jason Eakes, Sarah Moore, Kerry Hennessy, Ann Lin

**Affiliations:** a USF Health Morsani College of Medicine, University of South Florida, Tampa, Florida; b Department of Dermatology and Cutaneous Surgery, USF Health Morsani College of Medicine, University of South Florida, Tampa, Florida

**Keywords:** acute generalized exanthematous pustulosis, AGEP, drug reaction, naltrexone

## Introduction

Acute generalized exanthematous pustulosis (AGEP) is characterized by the rapid development of nonfollicular sterile pustules on an erythematous base, most often after drug exposure to anti-infectious agents.^1^ It can occur at any age but is most commonly seen in middle aged to older adults, with a female predominance.^2^ Common offenders include antibiotics, antifungals, antimalarials, and diltiazem, and AGEP eruption typically develops within 3 days of drug administration.^2^ Here we present a case of naltrexone-induced AGEP—an agent that has been previously reported to induce pustular psoriasis, urticaria, and eosinophilic pustular folliculitis.^3^, ^4^, ^5^

## Case report

**Fig 1:**
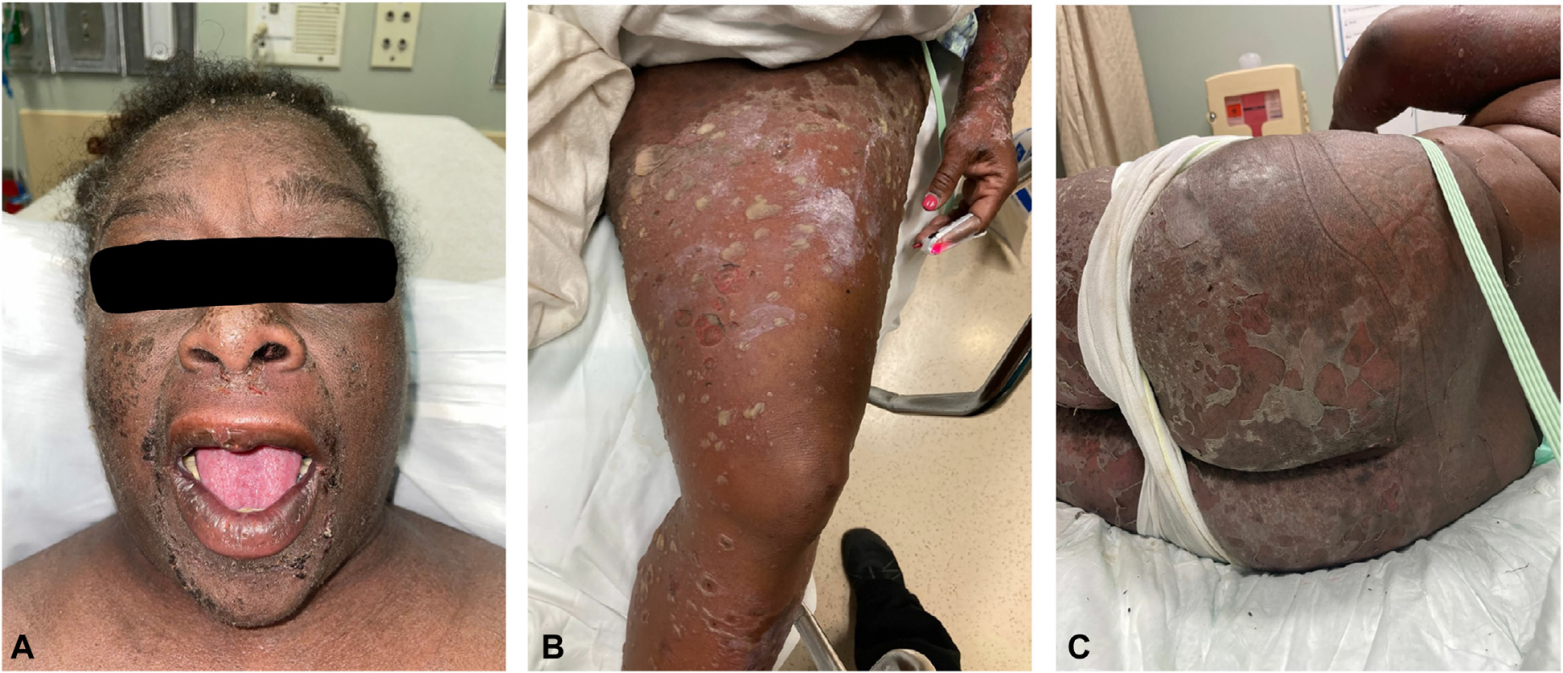
Acute generalized exanthematous pustulosis.

A 55-year-old woman with type 2 diabetes mellitus, hypertension, and hyperlipidemia presented to the emergency room as a hospital transfer with an 11-day history of a pruritic, painful, blistering rash on the trunk, face, and extremities without mucosal involvement. Her history was pertinent for naltrexone administration for treating alcohol use disorder 3 days before the development of the rash. The patient denied any other new prescription, over the counter, illicit, or supplemental drug use. Before 11 days, the patient was admitted to an outside facility for fever and perioral rash, where she received antibiotics and steroids for 1 week before being discharged. Further, the patient presented to a second outside facility 2 days later with a new vesiculobullous eruption and progression of the rash to her trunk and extremities. She was subsequently transferred to our facility for evaluation. On physical examination, she was afebrile and tachycardic and found to have diffuse erythema with bullae and pustules with intermittent dependent pus lakes, hyperpigmented scaly plaques on the face extending to the scalp, several erythematous papules coalescing into semiannular plaques, and desquamation that was most pronounced on the extremities (Fig 1). The patient had a negative Nikolsky sign, negative Asboe-Hansen sign, no palmoplantar involvement, and no oral, genital, or ocular mucosal involvement. Total body surface area involved was 40%, with 6% open. Laboratory results were significant and showed that the patient had leukocytosis with a total leukocyte count of 26.78 × 10^3^/mcL, neutrophilia with a total neutrophil count of 24.14 × 10^3^/mcL, eosinophilia with a total eosinophil count of 0.89 × 10^3^/mcL, and a negative antinuclear antibody titer.

**Fig 2:**
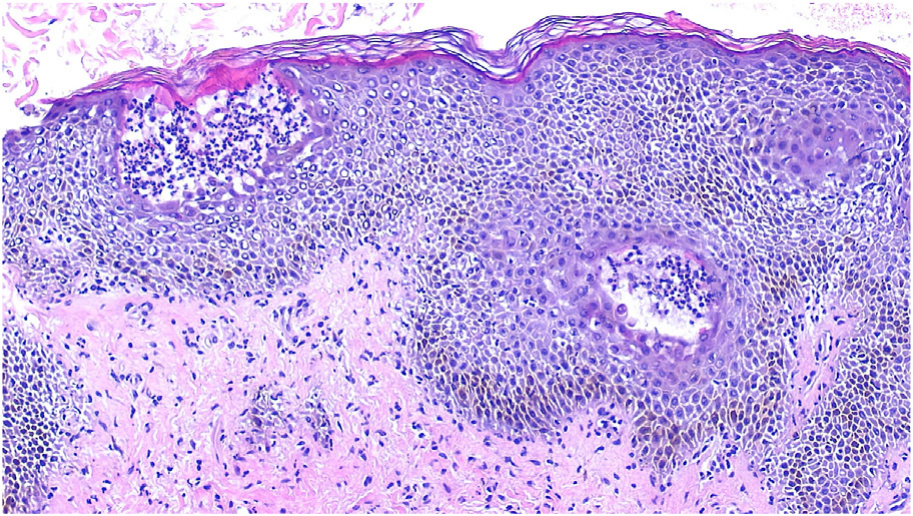
Punch biopsy with hematoxylin and eosin stain.

A 4-mm punch biopsy was performed, which revealed neutrophilic dermatitis with subcorneal pustules and a superficial and mid-dermal perivascular and interstitial infiltrate composed of lymphocytes, eosinophils, and scattered neutrophils (Fig 2). There was no evidence of epidermal necrosis or fungal elements and direct immunofluorescence was negative, favoring a diagnosis of AGEP. Other subcorneal blistering diseases were considered, including generalized pustular psoriasis, staphylococcal scaled skin syndrome, Sneddon-Wilkinson disease, AGEP, pemphigus foliaceus, and IgA pemphigus. Ultimately, the findings of negative immunofluorescence, presence of eosinophils, and overall clinical picture were most consistent with AGEP. Unfortunately, the final pathology did not present for several days, and given the differential diagnosis of AGEP vs pustular psoriasis and severity of illness, the patient was admitted to the burn unit where she was started on cyclosporine 3 mg/kg, and naltrexone was discontinued. The patient’s condition improved significantly with supportive care and cyclosporine, allowing for discharge on hospital day 6. Cyclosporine was discontinued at discharge, and the patient had complete resolution of the eruption at the 2-week follow-up.

## Discussion

The largest retrospective case series review to date of 340 patients diagnosed with AGEP found that approximately 85% of the AGEP cases were drug induced.^2^ AGEP has also been reported with various infections, radiocontrast media, and arachnid bites, but many cases are idiopathic.^2^^,^^3^^,^^6^ Since a detailed investigation in our patient did not indicate any previously reported causes of AGEP, we propose naltrexone use as the etiology based on the clinical timeline and characteristic eruption. Interestingly, the use of similar opioid pharmacological agents have been reported with this condition.^7^

In 2021, Caldas et al presented a case of naltrexone-bupropion-induced pustular psoriasis proven via biopsy. However, subsequent patch testing with naltrexone alone did not illicit a clinically observable reaction. The authors concluded that naltrexone alone was not the causative agent, and subsequent biopsy favored pustular psoriasis over AGEP.^3^ Similarly, in 2022, Smith et al presented a case of naltrexone-induced cutaneous adverse reaction, but they concluded that AGEP was less favorable based on the patient presentation and favored eosinophilic pustular folliculitis proven via biopsy.^5^ Interestingly, in 2020, Ghazawi et al presented a case of AGEP with topical administration of lidocaine and 10% morphine, which was subsequently confirmed via patch testing.^8^ Given the shared μ opioid receptor interactions between morphine and naltrexone, this could contribute to the pathophysiology of AGEP development in both cases.

It is impossible to assess a causal-effect relationship between naltrexone and AGEP. However, the use of the Adverse Drug Reaction Probability Scale (Naranjo Algorithm) provided a score of >6, indicating that the AGEP reaction that developed after the administration of naltrexone was probable.^9^ This is because the reaction following drug administration that resulted in a recognizable and confirmed AGEP reaction could not be reasonably explained by other offenders or competing diagnoses, and the patient showed rapid improvement with withdrawal of the drug.

We present the case of a patient who developed AGEP 3 days after administration of naltrexone to raise awareness about this agent as a potential trigger and recommend monitoring of patients for rash development within 2 weeks of administration.

## Conflicts of interest

None.
